# Toxicity study of TEoS-DAZA, a chemical precursor for functional liver imaging with PET/CT

**DOI:** 10.1186/s41181-025-00342-x

**Published:** 2025-05-17

**Authors:** Julia Greiser, Beatrice Engert, Roman Föll, Robert Klopfleisch, Rebecca Steens, Marion Hecht, Martin Freesmeyer

**Affiliations:** 1 Nuclear Medicine Department, Jena University Hospital, Am Klinikum 1, 07747 Jena, Germany; 2 Inflamed Pharma GmbH, Winzerlaer Straße 2, 07745 Jena, Germany; 3 Preclinical Science—Föll, Mecklenburg and Partner GmbH, Althausweg 158, 48159 Münster, Germany; 4 Department of Veterinary Medicine, Institute for Veterinary Pathology, Free University of Berlin, Robert-Von-Ostertag-Straße 15, 14163 Berlin, Germany; 5 Vivo Science GmbH, Fabrikstraße 3, 48599 Gronau, Germany

**Keywords:** Radiopharmaceutical, Chemical precursor, Preclinical toxicological study, Microdosing, ICH M3 (R2), TEoS-DAZA, Hepatobiliary PET

## Abstract

**Background:**

*N*,1,4-Tri(4-ethoxy-2-hydroxybenzyl)-1,4-diazepan-6-amine (TEoS-DAZA), a novel radiopharmaceutical precursor for a liver-specific ^68^Ga-based diagnostic radiopharmaceutical, was tested for toxicity in rats to ensure its safe applicability and to fulfil the preclinical requirements in preparation of a clinical study. The study was performed according to EMA draft Guideline on the non-clinical requirements for radiopharmaceuticals, as well as to the so-called microdosing approach of the ICH guideline M3 (R2).

**Results:**

This randomized study was conducted using Wistar rats. The test item was administered intravenously at three different dose levels, the vehicle solution was administered to a separate group as control. Toxicity assessment included a 24 h observation period in three dose groups, and a 14-day recovery period in the high dose group. Animals were monitored regarding clinical behaviour, bodyweight, food and water consumption, additionally undergoing modified IRWIN, grip-strength and beam-walking tests. Following euthanisation, extensive haematological and clinical biochemical parameters were analysed. Necropsy and histopathology were performed. There was no evidence to any test-item related adversities at any dose level. No delayed effects were identified in any animal at the end of the recovery phase. Some small, albeit significant changes in haematology and clinical biochemistry could not be related to the test item administration. The NOAEL of TEoS-DAZA was determined at 1.4 mg/kg bodyweight.

**Conclusions:**

Administration of a thousandfold clinical dose of TEoS-DAZA in rats did not cause any observable adverse events. An injectable solution of [^68^Ga]Ga-TEoS-DAZA containing 100 µg of the precursor is safe for clinical application to humans from the pharmacological point of view. Subsequent dosimetry studies need to be undertaken to reveal any radiation related toxicity.

**Supplementary Information:**

The online version contains supplementary material available at 10.1186/s41181-025-00342-x.

## Background

**Fig. 1:**
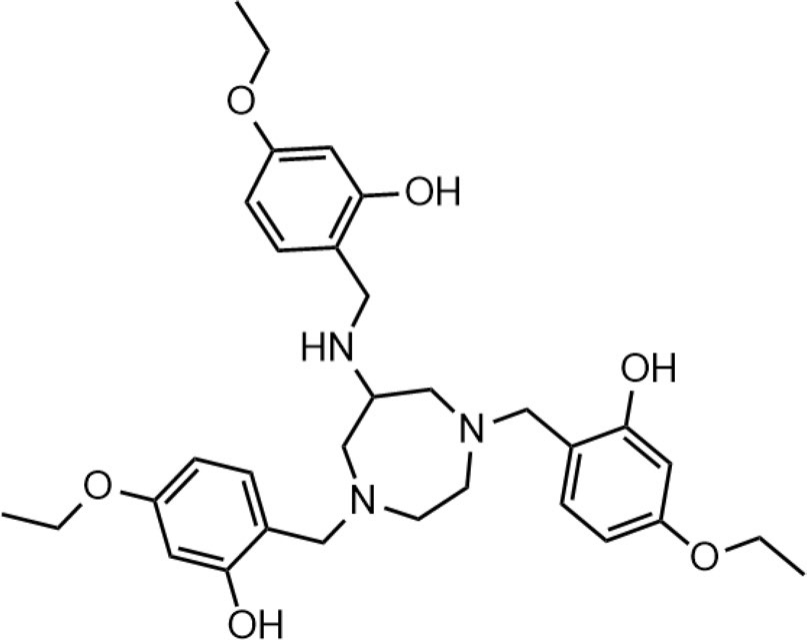
Chemical structure of TEoS-DAZA

*N*,1,4-Tri(4-ethoxy-2-hydroxybenzyl)−1,4-diazepan-6-amine (TEoS-DAZA, Fig. 1) is a recently developed compound from a group of novel chelators based on the heptacycle 1,4-diazepan-6-amine (DAZA) (Greiser et al. 2018). The nitrogen atoms of the DAZA cycle are alkylated by three functionalized hydroxybenzyl groups, thus forming a hexadentate chelator suitable for metal coordination. TEoS-DAZA was developed as chemical precursor for a radioactive metal complex, [^68^Ga]Ga-TEoS-DAZA, which is intended as a liver specific radiopharmaceutical for positron emission tomography (PET). [^68^Ga]Ga-TEoS-DAZA has proven very useful for liver-bile related diagnostics in several justified single applications, which so far were performed within the legal frame of the German Medicinal Products Act (AMG, §13, 2b) (Greiser et al. 2018, 2022; Freesmeyer et al. 2021, 2022, 2024). The aim of this toxicity study was to evaluate the nonclinical safety of the radiopharmaceutical formulation in relation to the legislative preclinical requirements according to the European Medicines Agency ‘s (EMA) scientific guideline „Non-clinical requirements for radiopharmaceuticals “, a complementary document draft to the ICH M3 (R2) guideline on „Non-clinical safety studies for the conduct of human clinical trials and marketing authorization for pharmaceuticals “ (CPMP/ICH/286/95) (European Medicines European Medicines Agency 2018; European Medicines Agency 2009; Koziorowski et al. 2017) In particular, this study aimed to provide information about potential adverse effects of the TEoS-DAZA precursor following intravenous injection. The results will support the conduction of future clinical trials.

Regarding pharmacological safety studies radiopharmaceuticals are special due to the non-labeled precursor usually being present in large molar excess over the radiopharmaceutical active ingredient (Koziorowski et al. 2017) Therefore, the precursor should be used for preclinical toxicity studies (Koziorowski et al. 2017) Since in most radiodiagnostic formulations, the precursor content usually does not exceed 100 µg per single administration, a dose that falls within the range of the so-called „microdosing approach 1 “ outlined in ICH M3 (R2), general safety consideration for radiopharmaceuticals often focus on dosimetry, i.e. radiation-related toxicity studies, while reports on the preclinical precursor toxicity are sparse (European Medicines Agency 2018, 2009) It was recommended by the Radiopharmacy Committee of the EANM (European Association of Nuclear Medicine) to make a risk assessment for each compound to evaluate which toxicological studies are needed and useful (Koziorowski et al. 2017) A final injectable formulation of [^68^Ga]Ga-TEoS-DAZA contains 100 µg of the precursor TEoS-DAZA, thus bordering the limits of the microdosing concept. Also, TEoS-DAZA is a novel aminophenolate not easily comparable to established radiopharmaceutical precursor ligands, like the aminocarboxylate DOTA (1,4,7,10-tetraazacyclododecane-*N,N',N",N"'*-tetraacetic acid). Aiming to conduct a first time clinical trial, our risk assessment concluded that a toxicity study with the precursor should be carried out as recommended by the ICH guideline M3 (R2) (European Medicines Agency 2018, 2009).

The guideline recommends testing a thousand-fold excess of a defined clinical single dose of the test item on a mg/kg bodyweight basis in a test species using the intended route of administration (European Medicines Agency 2018, 2009; Koziorowski et al. 2017) A study in one species is generally considered sufficient (European Medicines Agency 2018) Haematology, clinical chemistry, macroscopic evaluation at necropsy and histopathology data should be evaluated within 24 h following single administration, while a second group of animals should be monitored for 14 days to assess delayed toxicity and/or recovery (European Medicines Agency 2009) Following these requirements, this study was designed to test for potential toxic effects of the precursor TEoS-DAZA after a single intravenous injection followed by a 24 h observation period and a 14-days observation period for the recovery groups. The study was performed according to GLP principles in a certified test facility.

## Materials and methods

### Preparation of test item and vehicle solution

The test item solutions were handled and dosing solutions were prepared in biological safety cabinet for sterility. Injection solutions were prepared freshly on each injection day. 1.4 mg of the test item was dissolved in 50 μl DMSO, 500 μl of a PVP K25 (polyvinylpyrrolidone) solution (30% w/v, prepared by dissolving 9 g in 30 ml purified water) and 450 μl sterile saline (0.9%, w/v) for a final concentration of 1.4 mg/ml. The dilution of the stock solution to the high, medium and low dose concentrations was carried out with saline (0.9%, w/v) from the stock solution directly and not as dilution series in order to avoid thereof resulting deviations in concentrations. The vehicle solution was prepared in the same proportions of DMSO, PVP K25 (30% w/v) and saline as described for the stock solution.

### Test animals and animal husbandry

The study was conducted using specified pathogen free, nulliparous, non-pregnant rats (*rattus norvegicus*) of the Wistar Han IGS (Crl:Wi(Han)) strain (Charles River, Sandhofer Weg 7, 97,633 Sulzfeld, Germany). In total, 100 animals (50 males/50 females) were used, whereof 80 were selected for the main group, intended for monitoring of toxicity effects up to 24 h post injection, and 20 animals were selected for the recovery group, allowing for monitoring of delayed toxicity and recovery up to 14 days post injection. The animals were 8 weeks old at time of acclimatization. The body weight was 186–233 g (male) and 139–174 g (female). Upon arrival, the animals were visually checked for signs of ill health and abnormalities. The animals were acclimatized to the laboratory conditions for 7 to 9 days before the day of test item application.

All animals assigned to this study were housed in the same room throughout the study period. Animals were kept in a specified pathogen free housing exhibiting 15 air changes per hour and a temperature of 22 ± 3 °C, relative humidity at 30–70% and artificial lighting in a 12 h light/12 h dark setting with twilight phases, with permanent health monitoring of the barrier by sentinel animals (bedding sentinels) in accordance with FELASA recommendations (Mähler et al. 2014).

During the in-life phase, the rats were housed in groups of five animals per cage and sex, using open macrolon cages type 2000P (Tecniplast S.p.A., Italy) equipped with heat-treated aspen animal bedding (Tapvei Estonia OÜ, Harjumaa, Estonia). Wooden gnawing blocks and rat retreat (red-transparent polycarbonate, size 9 × 9 × 15 cm PLEXX B.V.) were used as cage enrichment. Animals were fed with maintenance diet for rats and mice (No. 1324 TPF, Altromin Spezialfutter GmbH & Co. KG, D-32791 Lage), ad libitum*,* and sterilized community tab water, ad libitum*.*

### Stratification, randomization and identification

This study was designed as a randomized controlled trial. Upon arrival at the test facility, rats were weighed individually and grouped into three weight clusters. Individual animals from the central clusters were placed consecutively into prepared cages until the cluster was depleted. Animals from the remaining clusters were caged alternately in the same manner. Prepared cardboard labels containing the dose group were riffled by hand and attached blindly onto the cages. Caged animals were identified by individual ear punches. Each cage was labelled with a card displaying the cage number, the study number, information on the animals and the respective dose group.

### Injection and dose calculation

For monitoring toxicity effects during 24 h post injection (main group), TEoS-DAZA was administered by intravenous, slow bolus injection to 10 male and 10 female rats each at the respective dose levels of 1.4 mg/kg (high dose); 0.2 mg/kg (medium dose) and 0.1 mg/kg (low dose) bodyweight, at a volume of 2.5 ml/kg bodyweight (Table 1). The individual dose volumes were calculated from the latest actual bodyweight data. Additionally, 10 male and 10 female animals received 2.5 ml/kg bodyweight of the vehicle solution as control. The day of administration was designated as day 1 (d1). The animals were anaesthetized 24 h (± 2 h) after the injection, blood was taken and the animals were euthanized in a CO_2_-enriched atmosphere, respectively. For monitoring delayed toxicity and recovery, an additional group of 10 animals (5 males/5 females) was treated with the test item dose level of 1.4 mg/kg (recovery group). Further 10 animals (5 males/5 females) received the vehicle solution as control; all other parameters were identical. The animals were observed for 14 days post injection before sacrifice. The number of test animals per group and the time points for analysis (24 h and 14 d) were chosed based on the recommendations in the ICH guideline M3 (R2) (European Medicines Agency 2009).

**Table 1:** Study design and dose groups

Group	Dose level [mg/kg]	Dose volume [ml/kg]	Dose concentration [mg/ml]	Number of animals	
				Main group (24 h)	Recovery group (14 days)
1	1.4	2.5	0.56	10 m/10 f	–
2	0.2	2.5	0.08	10 m/10 f	–
3	0.1	2.5	0.04	10 m/10 f	–
4	0 (vehicle control)	2.5	0	10 m/10 f	–
5	1.4	2.5	0.56	–	5 m/5 f
6	0 (vehicle control)	2.5	0	–	5 m/5 f

### Monitoring of in-life period

During the in-life phase, all animals were monitored for general clinical signs, clinical and behavioural abnormalities, bodyweight, as well as food and water consumption, as given in Table 2. Food and water consumption was determined summarily for each cage and divided by the number of animals per cage. Cage-side observations to detect signs of illness or reactions to treatment, moribund animals or fatalities were conducted at least once daily throughout the in-life phase. At the end of the in-life phase, blood samples from all animals were collected to acquire data on haematology and clinical biochemistry. Subsequently, all animals were sacrificed and examined by gross necropsy. Tissues and organs were preserved and processed for histopathological examination. Weights of selected organs (spleen, liver, adrenal glands, kidneys, thymus, heart with and without lungs, brain, testis, epidymis, prostate and thyroid with parathyroid) were recorded. The choice of organs was based on the EMA guideline on repeated dose toxicity (European Medicines Agency 2010).

**Table 2:** Parameters observed and documented during the in-life period and test frequency

Viability/mortality	Daily
General clinical signs/behavior	Daily
Detailed clinical signs (modified IRWIN test)	Once before administration start, main group: d1 at peak concentration*, recovery group: d1 at peak concentration*, d8 and on the day before necropsy
Grip-strength and beam-walking tests	Main group: d1 at peak concentration* Recovery group: on the day before necropsy
Bodyweight; food/water consumption	d1 (before application) and d2; additionally for recovery group: d8 and d15
Blood sampling	On the day of necropsy

^*^As serum peak concentration time point, 15 min p.i. were chosen, since earlier time points were not suitable for carrying out the modified IRWIN test

### Detailed clinical signs (modified IRWIN test)

Monitoring of individual detailed clinical signs was performed employing a modified IRWIN test on a time schedule described above (Irwin 1968). Findings were noted in the following categories: Appearance (general status, physiology, autonomic functions, neurology, tonus), motoric/exploration behaviour, excitation, abnormal behaviour. The test was performed on the top of an elevated standard arena (42 × 26 × 14 cm) outside the cage. Endpoints were evaluated with a scoring system and summarized in diagnostic clusters. For each cluster standard values were defined to discriminate between normal and altered clinical signs.

### Grip-strength and beam-walking test

Grip strengths and beam walking tests were recorded in order to assess muscle strength and motor coordination (Goldstein and Davis 1990) The tests were performed on d1, 15 min p.i. for main group animals and on d14 for the recovery groups. For the assessment of grip strength, untrained rats gripping a small grid attached to a grip strength meter (TSE 303500. vivo Science ID INV284, TSE Systems GmbH, Germany) were manually pulled away from the grid. The maximum read-out (in grams) was denoted as the individual rat’s grip strength. For the beam-walking test, animals were placed on a small, horizontal, wooden bar (5 cm × 130 cm width/length). The coordination and balance while crossing the bar and the placing and -if so- faults of the foot placement on the bar was rated in a 7-point scoring system as depicted in Table 3.

**Table 3:** Beam-walking scores

Observations/findings	Score
The animal is able to place all limbs on the bar but refuses to cross the bar	–
The animal is not able to place all limbs on the bar	1
The animal places all limbs on the bar and keeps the balance for at least 5 s	2
The animal crosses the bar with limbs dragged behind (footslips)	3
The animal crosses the bar (dragged limbs) and places at least the limb(s) once on the bar	4
The animal crosses the bar, more than half of the steps are footslips	5
The animal crosses the bar, less than the half and more than two of the steps are footslips	6
The animal crosses the bar with not more than two footslips	7

A score of 6 or 7 points was regarded as normal. In some instances, it is natural behaviour even for untreated, healthy rats to refuse a crossing of the bar at all, resulting in no scoring points. In addition, the latency time (time until the animal reaches the target box with its nose; up to 90 s) was recorded. Latencies of > 90 s are assigned to animals falling from the bar. Likewise, animals that refuse to cross the bar are rated with a latency time of > 90 s.

### Blood sampling for haematology and clinical biochemistry

Blood sampling was performed for all experimental animals at the end of the in-life phase, i.e. day 2 (d2, main) or day 15 (d15, recovery). Approx. 2000 μl blood were taken from the retro-orbital vein plexus of each animal. Animals were anaesthetized before sample collection. The blood samples were divided to the following tubes:

- 500 μl into an EDTA-coated tube for haematology.

- 500 μl into a lithium heparin-coated tube for clinical biochemistry.

- 500 μl into a sodium citrate-coated tube for blood clotting time.

The EDTA-tube was filled directly after bleeding to avoid coagulation. The lithium heparin blood sample and the sodium citrate sample were used to assess plasma biochemistry and blood clotting time. The EDTA blood and the plasma samples were used to analyse parameters on haematology and clinical chemistry as well as blood clotting time. Blood analysis was performed within 8 h after bleeding at maximum.

### Necropsy and histology

The in-life phase was terminated on d2 (main group) or d15 (recovery group). All animals were sacrificed by asphyxiation in a CO_2_-enriched atmosphere and subsequent exsanguination. A full macroscopic examination was performed. All occurring lesions were noted. Data were recorded on checklists for each individual animal. During necropsy, from all experimental animals, the organs were preserved in the appropriate fixative/s. Selected organs were trimmed of any adherent tissue and their wet weight was taken as soon as possible after dissection. Paired organs were weighed individually (if applicable). Modified Davidson solution was used for testis and epididymis; Davidson solution was used for the eyes. All other organs/tissues were fixed in phosphate-buffered formalin. The tissues were embedded in paraffin wax, sectioned, and stained with hemalaun and eosin.

### Statistical analysis

Spreadsheet calculations were performed using validated Microsoft® Excel® sheets. The bodyweight was documented for each individual animal. Food and water consumption were documented sorted by experimental groups and sex. For each experimental group, means and standard deviations were calculated, if applicable.

The arithmetic mean, standard deviation and median were calculated for all grouped numerical data originating from the monitoring of gross pathology (organ weights) and parts of the haemogram. Where appropriate, detailed column statistics were applied (minimum/maximum data, 25% and 75% quartiles, standard error, upper and lower confidence interval 95%).

For basic analysis, the respective test item group was compared to the vehicle group by assessing of statistical significance using a two-tailed unpaired Student´s t-test. For all calculations, the significance level was set to 0.05. In case the basic analysis revealed a statistical significance, additional inductive statistical analysis was applied, according to the decision tree as given in the supplementary information (Fig. S.1). Except for individual blood parameters (monocytes, eosinophils, basophils; only descriptive statistics) it was assumed that blood data collected in the present study are metrically scaled and normally distributed (Gaussian). Most statistical hypotheses in this study were best characterized as “many to one”– comparing a vehicle control vs. three treatment groups, respectively. Therefore, the adequate inductive statistical analysis methods were one-way ANOVA (analysis of variance) followed by a post-hoc Dunnett´s t-test (e.g. for organ weights), or two-way ANOVA followed by a post-hoc Bonferroni test (for bodyweight analysis). For all calculations, the significance level was set to 0.05. These further inductive statistics was calculated using Graph Pad Prism, Version 9 (Dotmatics, Boston, MA, USA). Whenever the term “significant” is used in this article, it stands for “statistically significant”. Non-parametric data of the modified IRWIN test results were listed as a complete overview of individual observations. Since there were no abnormalities detected in any test animal, further statistical analysis of the modified IRWIN test results was not necessary.

## Results

### General and detailed monitoring of clinical signs and behaviour

During the entire in-life phase, no test item related abnormalities regarding general clinical signs were observed in any of the experimental animals. No animal of the main or recovery dose groups died or had to be prematurely euthanized during this study for humane reasons. Endpoints assessed within the scope of the modified IRWIN test such as health status and overall physiological condition including appearance, motor activity, excitation, stereotypies, and abnormal behaviour revealed no test item-related changes, neither during treatment period nor during recovery phase. An elevated muscle tonus was observed for one female animal before test item administration and was therefore rated as an inherent reaction to the new handling situation (first modified IRWIN test).

### Grip-strength and beam-walking test

Detailed data on the results of grip-strength and beam-walking test are given in the supplementary information (Tables S.1–S.4). Basic statistical analysis (Student’s t-test, two-tailed, unpaired) of the results of the grip strength returned no differences between the test item treated animals and the respective controls (Table 4). For the main groups, approximately 15 min after test item administration and for the recovery group during the last week of the recovery phase, the grip-strength values of both the respective male and female high, medium and low dose groups did not significantly differ when compared to the corresponding vehicle group.

**Table 4:** Results of grip-strength and beam-walking tests

Dose	Parameter	Main group (m)	Main group (f)	Recovery group (m)	Recovery group (f)
1.4 mg/kg (high)	Grip strength range [g]	1039.1 ± 104.3	1028.5 ± 84.6	1356.8 ± 76.3	1197.2 ± 65.1
	Beam walking score	6.9 ± 0.3	7.0 ± 0.0	7.0 ± 0.0	7.0 ± 0.0
0.2 mg/kg (medium)	Grip strength range [g]	1029.5 ± 151.5	1017.4 ± 53.1	–	–
	Beam walking score	6.9 ± 0.3	7.0 ± 0.0	–	–
0.1 mg/kg (low)	Grip strength range [g]	1065.6 ± 104.2	1066.3 ± 132.9	–	–
	Beam walking score	7.0 ± 0.0	7.0 ± 0.0	–	–
Vehicle (control)	Grip strength range [g]	1058.3 ± 153.9	1049.5 ± 114.9	1291.8 ± 179.6	1104 ± 95.4
	Beam walking score	7.0 ± 0.0	7.0 ± 0.0	7.0 ± 0.0	7.0 ± 0.0

The beam-walking scores of all animals were evenly distributed over all dose groups and the vehicle control group, both male and female, showing only slight differences in their respective latency periods. The noted differences were within the normal range of variability found among healthy animals.

### Body weight and food/water consumption

**Fig. 2:**
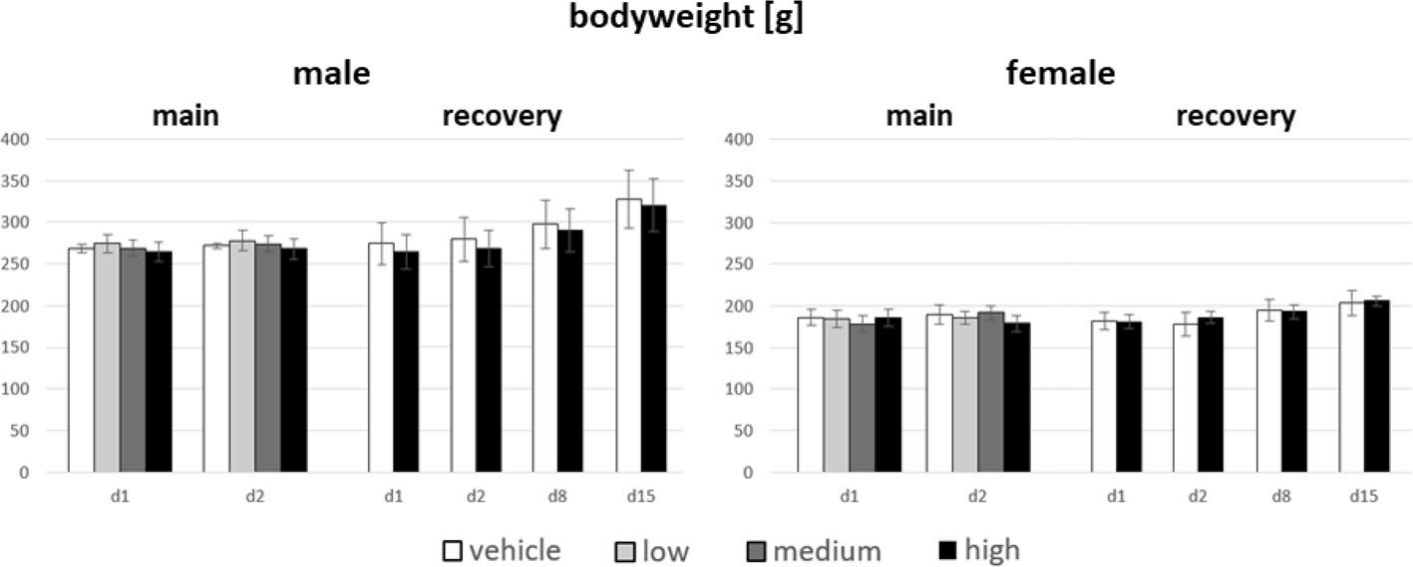
Mean absolute bodyweight of main and recovery group animals, given in g, sorted by dose groups, sex and day of measurement (n = 10). Means and standard deviations of each group are given

Detailed data on bodyweight and consumption values are given in the supplementary information (Tables S.5–S.16). The mean bodyweights and bodyweight gains observed in male and female experimental animals of both the study main groups and recovery groups (Fig. 2) were within normal range for rats of this sex, strain and age throughout the study periods of treatment and recovery. There was an increase in bodyweight during the recovery period, which was also observed in the control group. In summary, no significant differences regarding absolute bodyweight and bodyweight gain were detected in the main groups and in the recovery groups during the course of the treatment period.

**Fig. 3:**
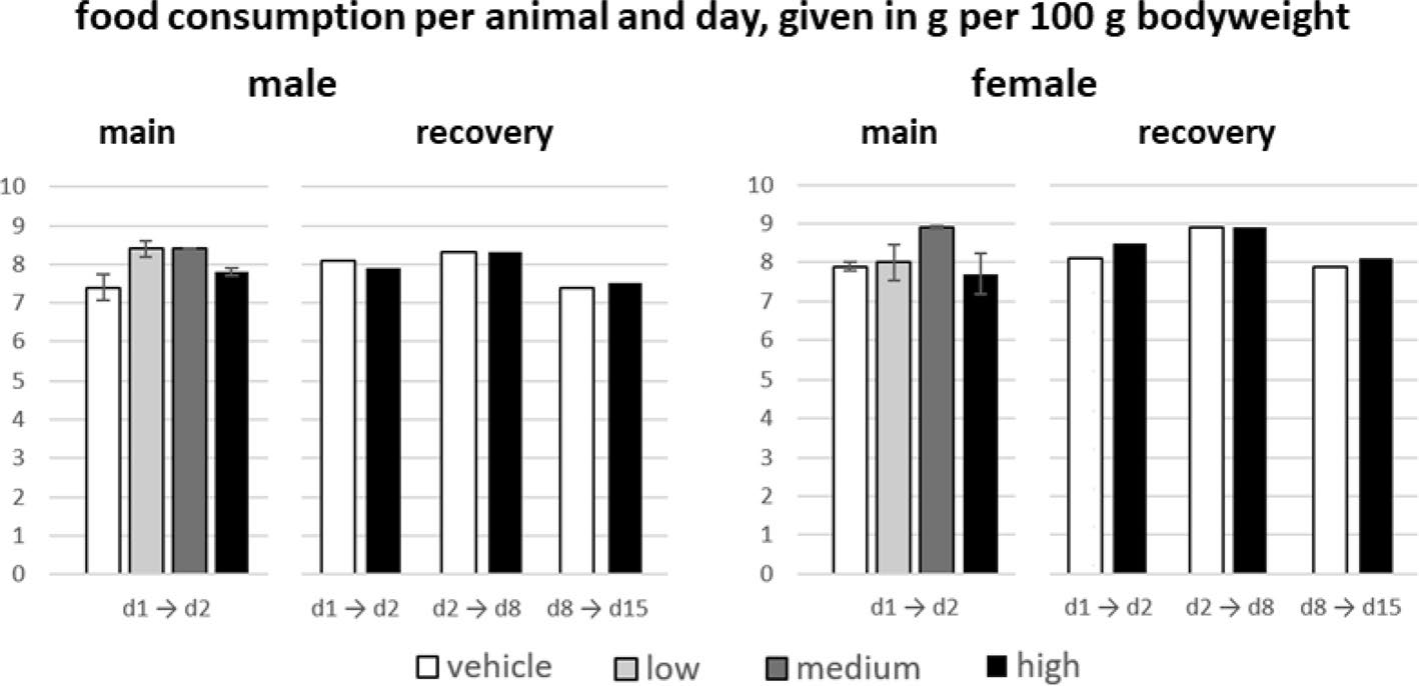
Mean food consumption per animal and day, given in g per 100 g bodyweight, sorted by dose groups, sex and observation period. Values were determined per cage (n = 2 for main groups, n = 1 for recovery groups) and subsequently divided by the number of animals per cage

**Fig. 4:**
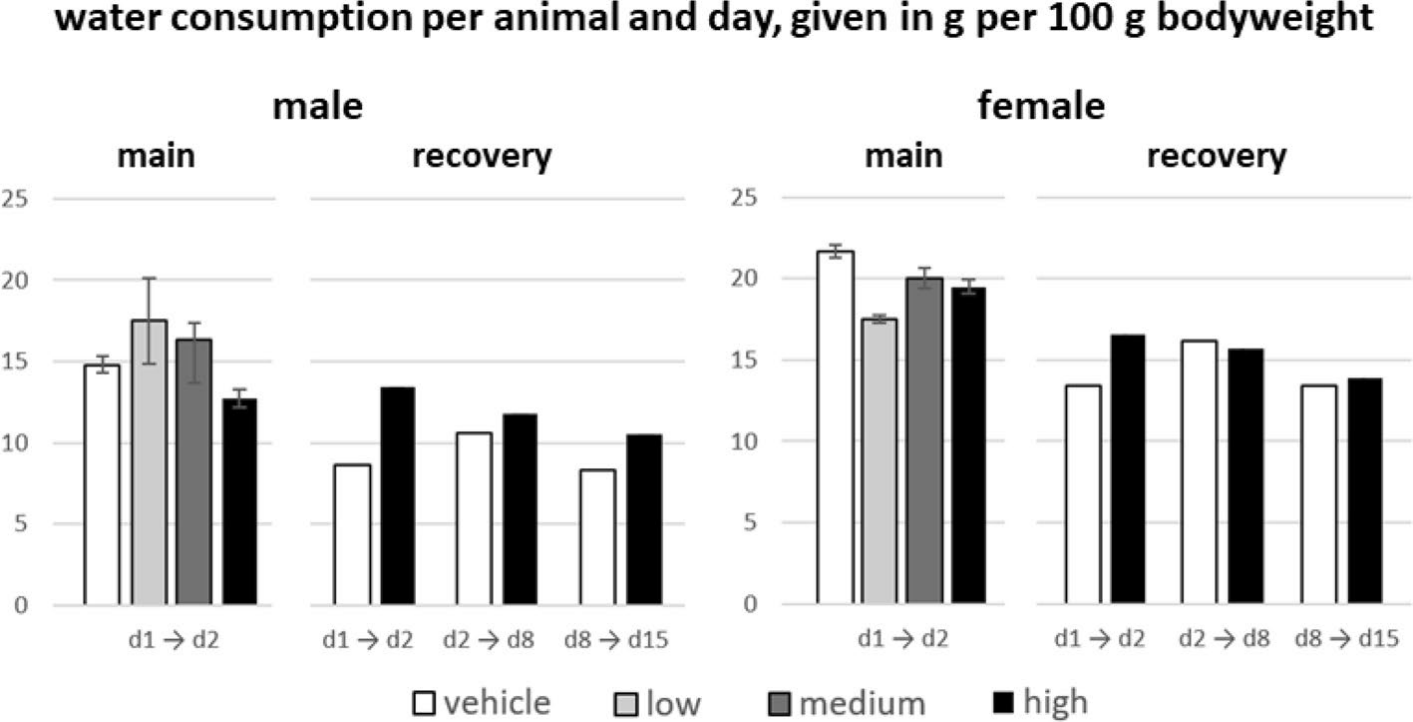
Mean water consumption per animal and day, given in [g per 100 g] bodyweight, sorted by dose groups, sex and observation period (n = 10 for main groups, n = 5 for recovery groups)

The food and water consumption were determined as total consumed amount in [g] per cage and was then calculated as [g] per animal and day. This value was set in relation to the animal bodyweight (per 100 g) in order to take into account the bodyweight change over time. Due to the number of biological replicates in the main groups (n = 10, kept in 5 animals per cage) the consumption values were calculated as a mean ± standard deviation for each cage (n = 2). In the recovery groups each of the four experimental groups (male/female, high dose/vehicle; n = 5 animals) was kept in one cage, respectively. Therefore, there was only one value of total consumed amount per recovery group with no mean value and therefore no standard deviation. Accordingly, only the values given for the main groups exhibit a standard deviation shown as error bars in Figs. 3 and 4.

Compared to concurrent controls, the average food consumption per 100 g bodyweight in the main groups was slightly increased (6% to 14%) in male animals of all dose groups during the 24 h post injection, while in females there was a slight decrease noted for the high dose group and an increase in the medium and low dose groups. The average food consumption in male and female animals of the recovery groups was within a variability in range of ± 10%, compared to the respective controls. The consumption values in the recovery vehicle groups in the first week (d2 → d8) and second week (d8 → d15) exhibited a variability compared to the values on the first day (d1 → d2) of ± 10%, indicating that the variability of some dose groups is comparable to the variability within the controls alone.

Compared to concurrent controls, the average water consumption per 100 g bodyweight in the main test item treated animals during the 24 h post injection was slightly decreased (−14%) in male high dose groups and increased (up to 18%) in male medium and low dose groups, while in females the low dose group exhibited the lowest consumption (− 19%, Fig. 4). The consumption values in the recovery groups were increased most notably during the first 24 h of the in-life phase (56% male, 24% female). Compared to the main group animals, such an increase was not observed for the high dose groups. In general, interpretations about the influence of the test item on the consumption values are hardly possible, given that the values are generated from only one data set per cage and taking into account the strong variabilities between the respective vehicle groups alone, e.g. water consumption between main and recovery period d1-d2 (males: 42%, females: 38%), or between recovery period d2-d8 and recovery period d1-d2 (females: 21%, males: 23%).

### Haematology and clinical biochemistry

All haematological and clinical biochemistry parameters are provided in detail in the supplementary information (Tables S.17–S.27). Only parameters exhibiting significant changes compared to the vehicle control group according to both, basic and additional inductive statistical analysis, are given in Table 5.

**Table 5:** Summary of significant observations in haematology and clinical biochemistry

	Affected dose group result	Vehicle result	Unit
*Main, male*			
Alkaline phosphatase (AP)	Medium: 323.0 ± 82.6	247.0 ± 48.7	U/L
Cholesterol (CHOL)	Medium: 1.538 ± 0.191	1.890 ± 0.315	mmol/L
	Low: 1.482 ± 0.209		
Globulin (GLOB)	High: 25.14 ± 0.772	23.40 ± 1.594	g/L
	Low: 24.94 ± 1.455		
Total protein (PROT)	High: 60.20 ± 1.476	57.60 ± 2.459	g/L
	Low: 60.00 ± 2.309		
*Main, female*			
Hematokrit (HKT)	Medium: 0.40 ± 1.01	0.42 ± 0.01	L/L
Mean corpuscular hematocrit (MCHC)	Medium: 34.6 ± 0.60	34.0 ± 0.49	g/dL
Triglycerides (TRIG)	High: 0.966 ± 0.367	1.374 ± 0.301	mmol/L
Urea (HST)	High: 6.49 ± 0.594	7.36 ± 1.120	mmol/L
	Medium: 6.46 ± 0.505		
Potassium (K)	Low: 3.90 ± 0.170	3.76 ± 0.158	mmol/L
Albumin/globulin ration (A/G)	Low: 1.39 ± 0.057	1.37 ± 0.048	g/L
*Recovery, female*			
Calcium (CA)	High: 2.59 ± 0.033	2.66 ± 0.026	mmol/L

In males of the main group, enhanced inductive statistical analysis revealed four significant changes in clinical biochemistry parameters. The alkaline phosphatase (AP) level was increased in the medium dose animals, the cholesterol (CHOL) levels was reduced in the medium and low dose animals and the globulin (GLOB) and total protein (PROT) levels were increased in the high and low dose animals, respectively (supplementary information, Table S.19). The changes in AP were solitary; no changes in other liver enzymes (e.g., aspartate aminotransferase) or correlating values such as bilirubin were detected. Changes in globulin and total protein, albeit statistically significant, were small between test item and control groups.

In females of the main group, inductive statistical analysis revealed a reduction for the hematocrit (HKT) and, as a result, an increase for the mean corpuscular haematocrit (MCHC) for the medium dose animals (supplementary information, Table S.22). Both changes, albeit statistically significant, were small between test item and control groups. In addition, the correlating values for the erythrocyte concentration and the haemoglobin value were not changed. Furthermore, no microscopic correlates were seen in the histopathological evaluation of the animals. Also, enhanced inductive statistical analysis revealed four significant changes in clinical biochemistry parameters: The triglycerides (TRIG) level was decreased in the high dose animals, the urea (HST) levels was reduced in the high and medium dose animals and the potassium (K) level and the albumin/globulin ration (A/G) were increased in the low dose animals.

In males of the recovery group, no significant differences were observed for the test item treated groups, when compared to the vehicle control group. In females of the recovery group, decreased values in calcium (CA) concentration were found for the high dose animals in comparison to the vehicle control group (supplementary information Table S.27).

### Necropsy

Reddened and/or enlarged lymph nodes (mandibular, inguinal and axillar) were noticed in animals of both sexes and of all dose groups and the vehicle control groups within the main group (Table 6). For one seminal vesicle, a reduction in size was found in one male animal of the high dose group. One male animal of the low dose group had an enlarged lung with dark-coloured apices. The left medial liver lobe was reduced in size in two female animals of the vehicle group. These findings were not dose-dependent and not associated with a microscopic finding and are thus considered as non-adverse background lesions or individual anatomical variation in these animals according to the histopathology results. Fluid was observed in the uterus of four females (main group) of the medium dose group and one female of the low dose group. This finding was correlated with an oestrous/early oestrous/proestrous phase of the oestrous cycle and thus a normal, non-adverse finding in the respective animals.

**Table 6:** Necropsy findings of animals, listed for all dose groups of the main groups (male and female) and for the high dose group (male) of the recovery group, since there were no findings in the female recovery groups (high dose and vehicle). Findings are given as number out of 10 for main groups and as number out of 5 for the recovery group

	Main—male				Main—female				Rec.—male
Dose group	High	Med	Low	Veh	High	Med	Low	Veh	High
Lymph node axillar: reddened	NAD	NAD	1	NAD	NAD	NAD	1	NAD	1
Lymph node mandibular: reddened	NAD	1	1	NAD	1	NAD	NAD	NAD	NAD
Lymph node inguinal: reddened	NAD	NAD	NAD	NAD	NAD	1	NAD	1	NAD
Lymph node inguinal: enlarged	NAD	NAD	NAD	NAD	1	NAD	1	NAD	NAD
Lung: enlarged, apices coloured dark red	NAD	NAD	1	NAD	NAD	NAD	NAD	NAD	NAD
Liver: medial liver lobe reduced in size	NAD	NAD	NAD	NAD	NAD	NAD	NAD	2	NAD
Seminal vesicle: reduced size	1	NAD	NAD	NAD					NAD
Uterus: filled with liquid					NAD	4	1	NAD	

Veh, vehicle; med, medium; NAD, no adversity detected; rec., recovery

In the recovery groups, there was a reddened axillar lymph node in one male animal of the high dose group. There were no findings in the female recovery groups (high dose and vehicle). In one male animal of the vehicle control group, there was yellow tissue observed between pancreas and stomach. This finding only appeared in this vehicle group animal and never in dosed animals.

### Organ weights

Detailed data on organ weights are given in the supplementary information (Table S.28–S.36). Organ weights were determined in absolute values in [g] and as relative to the respective animal’s bodyweight. The absolute and relative values were then calculated as a mean of each experimental group (i.e. male/female, main/recovery dose groups). In males of the main group, the relative (but not absolute) organ weights of the liver were found significantly reduced in the medium, but not in high or low dose group compared to the appropriate vehicle control group (Table 7). In females of the recovery group, the relative (but not absolute) organ weights of the liver and the kidneys were found significantly reduced in the high dose group, while in females of the main group the relative (but not absolute) weight of the left kidneys was increased compared to the controls. Females of the main group also exhibited significantly reduced heart weights (relative and absolute) in the high and medium dose group, and increased weights of the spleen (relative and absolute) in the high and low dose group.

**Table 7:** Selected absolute (abs.) and relative (rel.) organ weights with values exhibiting significant differences compared to the vehicle control hightlighted in bold. Values are given as mean ± SD (n = 10 for main group, n = 5 for recovery group)

	Main—male				Recovery—male	
	High	Med	Low	Veh	High	Veh
Liver—abs. [g]	12.811 ± 1.130	12.646 ± 0.813	13.845 ± 1.214	13.447 ± 0.720	14.946 ± 1.921	15.614 ± 2.170
Liver—rel. [g]	4.783 ± 0.332	**4.617 ± 0.300**	4.984 ± 0.323	4.950 ± 0.232	5.584 ± 0.618	5.581 ± 0.457

	Main—female				Recovery—female	
	High	Med	Low	Veh	High	Veh
Liver—abs. [g]	8.660 ± 0.706	8.725 ± 0.390	8.440 ± 0.540	8.909 ± 0.658	8.993 ± 0.490	9.682 ± 0.861
Liver—rel. [g]	4.612 ± 0.286	4.555 ± 0.197	4.546 ± 0.279	4.697 ± 0.208	**4.836 ± 0.330**	5.438 ± 0.355
Kidney left—abs. [g]	0.814 ± 0.068	0.799 ± 0.038	0.761 ± 0.056	0.771 ± 0.057	0.766 ± 0.033	0.795 ± 0.065
Kidney left—rel. [g]	**0.433 ± 0.025**	0.417 ± 0.022	0.409 ± 0.022	0.407 ± 0.021	**0.412 ± 0.023**	0.446 ± 0.015
Kidney right—abs. [g]	0.825 ± 0.052	0.834 ± 0.059	0.785 ± 0.056	0.805 ± 0.060	0.810 ± 0.029	0.836 ± 0.059
Kidney right—rel. [g]	0.439 ± 0.013	0.435 ± 0.022	0.422 ± 0.020	0.425 ± 0.023	**0.436 ± 0.020**	0.470 ± 0.026
Heart—abs. [g]	**0.726 ± 0.052**	**0.733 ± 0.035**	0.777 ± 0.075	0.809 ± 0.083	0.808 ± 0.042	0.801 ± 0.054
Heart—rel. [g]	**0.387 ± 0.018**	**0.383 ± 0.018**	0.418 ± 0.032	0.428 ± 0.052	0.435 ± 0.028	0.452 ± 0.047
Spleen—abs. [g]	**0.431 ± 0.058**	0.448 ± 0.052	**0.420 ± 0.040**	0.383 ± 0.029	0.396 ± 0.059	0.436 ± 0.032
Spleen—rel. [g]	**0.229 ± 0.026**	0.234 ± 0.029	**0.226 ± 0.017**	0.203 ± 0.019	0.212 ± 0.028	0.245 ± 0.025

### Histopathological examination

A minimal non-adverse perivenous acute haemorrhage at the administration site as a result of the mechanical trauma caused by intravenous administration of the test item or vehicle solution was identified in animals of all main groups including the main vehicle groups (7 high dose group animals, 1 medium group animal, 10 low group animals, 15 vehicle group animals). Signs of haemorrhage at the administration site were not observed in recovery group animals. The lesion is thus reversible and non-adverse.

A minimal to moderate submucosal eosinophilic infiltration of the gastric mucosa was found in 12 high dose group animals, 17 medium dose group animals, 14 low dose animals and 17 animals of the vehicle group of the main phase of the study. In addition, similar infiltrates were found in 8 high dose and 10 vehicle dose animals of the recovery group. There was no observable dose dependency or increased incidence in the dosed groups. One animal of the medium dose group showed a mild congenital, non-adverse malformation (anomaly) of one adrenal gland. Female animals of all groups showed a normal distribution of oestrous cycle phases without any adverse findings.

## Discussion

This toxicity study of TEoS-DAZA was conducted in accordance to requirements for microdosing studies in the ICH guideline M3 (R2). A single injection of [^68^Ga]Ga-TEoS-DAZA in humans will contain a planned maximum clinical dose of the precursor TEoS-DAZA of 0.1 mg/person (Greiser et al. 2022) Based on an average human weight of 70 kg, this corresponds to a dose level of 0.0014 mg/kg. According to the guideline “Guidance for Industry: Estimating the Maximum Safe Starting Dose in Initial Clinical Trials for Therapeutics in Adult Healthy Volunteers, FDA, July 2005”, (Food 2005) the human equivalent dose (HED) of 0.0014 mg/kg was converted into an animal dose by multiplication with a conversion factor of 6.2 (based on body surface area) and an additional safety factor of 10, resulting in a test item dose level of 0.1 mg/kg per animal. In addition, a medium dose level of 0.2 mg/kg, corresponding to twice the maximum clinical dose applied to humans was administered to further assess toxicity. A dose level of 1.4 mg/kg corresponding to a 1000-fold clinical dose on a mg/kg basis for i.v. administration of an estimated human dose for intravenous injection based to the ICH guideline M3 (R2) was added as a high dose group. Wistar rats are commonly used and recommended to assess toxicity (Weber et al. 2011) The recommended volumes for intravenous bolus injections in rats are 1–5 ml/kg (Consortium 2016), accordingly a volume of 2.5 ml/kg was used as an injection volume in this study.

TEoS-DAZA is the first compound among a group of novel aminophenolates based on the heptacyclic macrocycle DAZA (Greiser et al. 2018) Known compounds closest in structural similarity can be considered heptacyclic aminocarboxylates, e.g. „AAZTA “(Travagin et al. 2021) and aminophenolate-carboxylate hybrids, like HBED (*N,N'*-bis(2-hydroxybenzyl)ethylenediamine-*N,N'*-diacetic acid), but reports on the safety of these compounds are sparse (Bergeron et al. 2002) Given its strong liver-targeting character, as indicated by PET studies using the ^68^Ga radiolabelled TEoS-DAZA (Greiser et al. 2022; Freesmeyer et al. 2022), adverse events related to liver toxicity of TEoS-DAZA might be expectable. Also, as a metal ion chelator, TEoS-DAZA may exhibit some adverse reactions based on metal ion coordination or metal ion sequestration from tissue. So far, studies on other phenolate-based chelators reported no ligand-related adverse events (Bergeron et al. 2002) Given the very small molar amounts of TEoS-DAZA that are administered in the clinical context, a similar safety profile can be expected.

In our study, no animal of the main and the recovery group exhibited abnormalities regarding general clinical signs. IRWIN test endpoints such as health status and overall physiological condition confirmed an absence of any test-item related changes. Grip strength values and beam-walking scores of all animals did not significantly differ compared to the corresponding vehicle group. The food consumption values exhibited variabilities of up to 14%, but given this relatively low elevation, the lack of dose dependence and the normal variability range between the vehicle groups alone (± 10%) this implicates that the food consumption values are with no relation to test item administration. Likewise, the water consumption values within the vehicle control groups alone already showed high variabilities of up to 42%, therefore the observed variations between dosed animals and control animals (up to 56%) are likely not related to test item administration, also given the fact that none of these observations were dose dependant. No delayed effects were identified in the animal recovery phase. Taken together, the noted changes are most likely with no relation to test item administration and no macroscopic effects were classified as adverse.

Microscopic observations, including haematology, clinical biochemistry, macroscopic evaluation at necropsy, organ weight and histopathology revealed some statistically significant changes (Tables 5 and 7). However, none could clearly be related to test item administration, as they occurred in low, medium and high dose groups with no visible dose dependency and since haematological and biochemical parameters were not associated with microscopic findings. The differences regarding the weights of liver, kidneys, heart and spleen in some experimental groups are not of pathological significance in view of the small extend of the respective changes in organ weights, and in view of a lack of microscopic correlates in the histopathological analysis. Most of the changes were small and likely due to natural variability or of purely statistical significance without biological relevance. The increase in alkaline phosphatase and simultaneous decrease in cholesterol in the medium dose male main group may indicate liver damage, also considering that in the same group the relative (but not absolute) liver weight was reduced. However, the lack of change in other parameters indicating liver damage (e.g., aspartate aminotransferase), the unremarkable histopathology and the lack of a dose-dependent occurrence puts a possible relation to the test item into perspective. The statistical significance is therefore considered as incidental with no adverse effect associated. The most common finding in the necropsy was fluid in the uterus of four female animals, which was correlated with an oestrous/early oestrous/proestrus phase of the oestrous cycle and thus a normal, non-adverse event. The other events were singular and spread throughout all dose groups including the vehicle control groups. Minimal to moderate submucosal eosinophilic infiltration of the gastric mucosa was commonly found in all groups of animals, including vehicle groups, with no test item related occurrence and is thus diagnosed as non-adverse background finding. No persistent effects were noted for any experimental group at the end of the recovery phase. In summary, no clear relation to test item dosing was identified in any of the microscopic parameters and no adverse event could be associated with the test item.

The most concentrated injected solutions, which were administered to the high dose group, contained 0.125 ml/kg DMSO and 1.25 ml/kg PVP K25 (30%), given that a total volume of 2.5 ml/kg was injected. Both substances are used regularly as excipients in pharmaceutical or cosmetic products and are well evaluated regarding potential toxicities (Kurakula and Rao 2020; Nair 1998) Both the FDAs “Guidance for Industry: Q3 C Tables and List, FDA, June 2017"and the EMAs ICH Q3 C (R9)„ Guideline on impurities: Guideline for residual solvents “ list DMSO as a class 3 solvent, i.e. solvents with low toxic potential to man (European Medicines Agency 2024; Food 2017) In mice an intravenous DMSO injection of 1.0 ml/kg was considered safe (Montaguti et al. 1994) In canines, a 1.0 ml/kg slow infusion of DMSO (10%) was investigated and found not to exhibit any adverse reactions with little to no effect on haematology or serum chemistry, except for a slight, albeit significant change in mean corpuscular haemoglobin and mean corpuscular volume (Ruble et al. 2006) Oral exposure of rats to DMSO (2%) in water for several days resulted in elevated plasma cholesterol and reduced activity of hepatic cholesterol 7α-hydroxylase, indicative of DMSO in high doses potentially affecting cholesterol and bile acid metabolism (Hassan 1987) Other studies found that DMSO may even exhibit liver protective properties when co-administered with hepatotoxic substances or procedures (Lind and Gandolfi 1999, 1997; Sahin et al. 2004).

PVP K25 is a linear polymer with a molar mass of about 30.000 Da (Kurakula and Rao 2020) PVP is generally considered a biologically inert and non-toxic substance (Kurakula and Rao 2020) At the given molar mass, retention of PVP K25 in the reticuloendothelial system is likely (Kurakula and Rao 2020) For this toxicity study in rats, PVP K25 was chosen for the preparation of the test item injection solutions due to TEoS-DAZAs very limited solubility in water, in order to reach the very high concentration required to realize a thousandfold excess TEoS-DAZA injectable solution. However, in a radiopharmaceutical formulation of [^68^Ga]Ga-TEoS-DAZA prepared in a clinical setting, the inclusion of DMSO and PVP K25 is not necessary as the precursor starting amount can be readily dissolved by using an aquous acidified ethanol solution (Greiser et al. 2022).

In summary, whenever significant changes were observed in our study, these were not dose-dependent but occurred in any of the low, medium or high dose group. There was no indication that the animals in the high dose groups, both in the main and the recovery groups, are affected by adverse events related to the high test item dose. Therefore, the no observed adverse events level (NOAEL) of TEoS-DAZA is identified as 1.4 mg/kg bodyweight, i.e. the highest dose applied in the present study.

## Conclusions

A single intravenous bolus injection of TEoS-DAZA to rats at dose levels of 1.4 mg/kg (high dose), 0.2 mg/kg (medium dose) and 0.1 mg/kg (low dose) was overall well tolerated without macroscopic or microscopic evidence for local or systemic toxicity. Some minor changes in haematological and biochemical parameters and organ weights were observed without being related to test item administration. In conclusion of evidence and under consideration of the results obtained from the histopathological examination, all findings noticed were not regarded to be associated with adversity or toxicity of the test item. No persistent effects were identified in any animal of the high dose groups at the end of the recovery phase. Thus, the NOAEL of a single intravenous injection of TEoS-DAZA is determined at 1.4 mg/kg bodyweight. Therefore, a radiotracer formulation containing a maximum of 0.1 mg of TEoS-DAZA given to a patient of 70 kg bodyweight (0.0014 mg/kg) corresponds to a total dose of ≤ 1/1000 th NOAEL, which is ten times lower than the recommended dose according to the EMA guideline and the FDA’s “Guidance for industry, Developing medical imaging drugs and biological products, Part 1: conducting safety assessments”, with both recommending the NOAEL be at least one hundred times greater than the maximal dose to be used in human studies (European Medicines Agency 2009; Koziorowski et al. 2017; Food 2005) To conclude, [^68^Ga]Ga-TEoS-DAZA radiotracer formulations are safe for clinical application in humans.

## Supplementary Information

Supplementary materials 1

## Data Availability

Detailed data and results of the toxicological investigations are given in the ESI. More extensive data can be made available from the corresponding author on reasonable request.
