# Supplementary material for: Toxicity study of TEoS-DAZA, a chemical precursor for functional liver imaging with PET/CT
Source: EJNMMI Radiopharm Chem. 2025 May 17;10:24. doi: 10.1186/s41181-025-00342-x (PMC12085735; doi:10.1186/s41181-025-00342-x)
Supplement: Supplementary file 1 — Supplementary materials 1 [file 41181_2025_342_MOESM1_ESM.docx]

**Electronic Supplementary Information**

**Toxicity Study of TEoS-DAZA, a chemical precursor for functional liver imaging with PET/CT**

Julia Greiser^1^, Beatrice Engert^2^, Roman Föll^3^, Robert Klopfleisch^4^, Rebecca Steens^5^, Marion Hecht^5†^, Martin Freesmeyer^1†^*

^1^ Jena University Hospital, Nuclear Medicine Department, Am Klinikum 1, Jena, D-07747, Germany

^2^ inflamed pharma GmbH, Winzerlaer Straße 2, Jena, D-07745, Germany

^3^ Preclinical Science – Föll, Mecklenburg & Partner GmbH, Althausweg 158, Münster, D-48159, Germany

^4^ Free University of Berlin, Department of Veterinary Medicine, Institute for Veterinary Pathology, Robert-von-Ostertag-Straße 15, Berlin, D‑14163, Germany

^5^ vivo Science GmbH, Fabrikstraße 3, Gronau, D-48599, Germany

^†^ These authors contributed equally.

* Correspondence: Martin Freesmeyer, martin.freesmeyer@med.uni-jena.de, Jena University Hospital, Nuclear Medicine Department, Am Klinikum 1, Jena, D-07747, Germany

## Table S1. In-life-data: grip strength and beam-walking test: males -main-

Asterisks denote significance levels: n.s.: not significant (p > 0,05); *: 0,05 ≥ p > 0,01; **: 0,01 ≥ p > 0,001; ***: p ≤ 0,001 (Student ́s t-test two-tailed, unpaired).

## Table S2. In-life-data: grip strength and beam-walking test: females -main-

Asterisks denote significance levels: n.s.: not significant (p > 0,05); *: 0,05 ≥ p > 0,01; **: 0,01 ≥ p > 0,001; ***: p ≤ 0,001 (Student ́s t-test two-tailed, unpaired).

## Table S3. In-life-data: grip strength and beam-walking test: males -recovery-

Asterisks denote significance levels: n.s.: not significant (p > 0,05); *: 0,05 ≥ p > 0,01; **: 0,01 ≥ p > 0,001; ***: p ≤ 0,001 (Student ́s t-test two-tailed, unpaired).

## Table S4. In-life-data: grip strength and beam-walking test: females –recovery-

Asterisks denote significance levels: n.s.: not significant (p > 0,05); *: 0,05 ≥ p > 0,01; **: 0,01 ≥ p > 0,001; ***: p ≤ 0,001 (Student ́s t-test two-tailed, unpaired).

## Table S5. In-life-data: body weight [g] and body weight change (%): males -main-

Asterisks denote significance levels: n.s.: not significant (p > 0,05); *: 0,05 ≥ p > 0,01; **: 0,01 ≥ p > 0,001; ***: p ≤ 0,001 (Student ́s t-test two-ailed, unpaired).

## Table S6. In-life-data: body weight [g] and body weight change (%) : females -main-

Asterisks denote significance levels: n.s.: not significant (p > 0,05); *: 0,05 ≥ p > 0,01; **: 0,01 ≥ p > 0,001; ***: p ≤ 0,001 (Student ́s t-test two-tailed, unpaired).

## Table S7. In-life-data: body weight [g]: males -recovery-

Asterisks denote significance levels: n.s.: not significant (p > 0,05); *: 0,05 ≥ p > 0,01; **: 0,01 ≥ p > 0,001; ***: p ≤ 0,001 (Student ́s t-test two-tailed, unpaired).

## Table S8. In-life-data: body weight change to day 1 [%]: males -recovery-

## Table S9. In-life-data: body weight change to week before [%]: males -recovery-

## Table S10. In-life-data: body weight [g]: females -recovery-

Asterisks denote significance levels: n.s.: not significant (p > 0,05); *: 0,05 ≥ p > 0,01; **: 0,01 ≥ p > 0,001; ***: p ≤ 0,001 (Student ́s t-test two-tailed, unpaired).

## Table S11. In-life-data: body weight change to day 1 [%]: males -recovery-

## Table S12. In-life-data: body weight change to week before [%]: females -recovery-

## Table S.13. In-life-data: food and water consumption [g]: males -main-

|  |  |
| --- | --- |

## Table S.14. In-life-data: food and water consumption [g]: females -main-

|  |  |
| --- | --- |

## Table S.15. In-life-data: food and water consumption [g]: males -recovery-

## Table S.16. In-life-data: food and water consumption [g]: females -recovery-

## Table S.17. Hematology: males -main-

Asterisks denote significance levels: n.s.: not significant (p > 0,05); *: 0,05 ≥ p > 0,01; **: 0,01 ≥ p > 0,001; ***: p ≤ 0,001. (Student ́s t-test two-tailed, unpaired); Median-Mean: mean deviation from the median [%]

## Table S.18. Clinical biochemistry: males -main-

Asterisks denote significance levels: n.s.: not significant (p > 0,05); *: 0,05 ≥ p > 0,01; **: 0,01 ≥ p > 0,001; ***: p ≤ 0,001. (Student ́s t-test two-tailed, unpaired); Median-Mean: mean deviation from the median [%]

## Table S.19. Inductive statistical analysis: hematology/ clinical biochemistry: males -main-

GraphPad Prism software was used for statistical analysis. Asterisks denote significance levels: n.s.: not significant (p > 0,05); *: 0,05 ≥ p > 0,01; **: 0,01 ≥ p > 0,001; ***: p ≤ 0,001 (Dunnett ́s post hoc t-test after 1-way ANOVA).

## Table S.20. Hematology: females -main-

Asterisks denote significance levels: n.s.: not significant (p > 0,05); *: 0,05 ≥ p > 0,01; **: 0,01 ≥ p > 0,001; ***: p ≤ 0,001. (Student ́s t-test two-tailed, unpaired); Median-Mean: mean deviation from the median [%]

## Table S.21. Clinical biochemistry: females -main-

Asterisks denote significance levels: n.s.: not significant (p > 0,05); *: 0,05 ≥ p > 0,01; **: 0,01 ≥ p > 0,001; ***: p ≤ 0,001. (Student ́s t-test two-tailed, unpaired); Median-Mean: mean deviation from the median [%]

## Table S.22. Inductive statistical analysis: hematology/ clinical biochemistry: females -main-

GraphPad Prism software was used for statistical analysis. Asterisks denote significance levels: n.s.: not significant (p > 0,05); *: 0,05 ≥ p > 0,01; **: 0,01 ≥ p > 0,001; ***: p ≤ 0,001 (Dunnett ́s post hoc t-test after 1-way ANOVA).

## Table S.23. Inductive statistical analysis: hematology/ clinical biochemistry: males/females main

Heteroscedastic data (pos. Bartlett‘s test for equal variances) were analyzed using Kruskall-Wallis Test and Dunn‘s Multiple Comparison Test

## Table S.24. Hematology: males -recovery-

Asterisks denote significance levels: n.s.: not significant (p > 0,05); *: 0,05 ≥ p > 0,01; **: 0,01 ≥ p > 0,001; ***: p ≤ 0,001. (Student ́s t-test two-tailed, unpaired); Median-Mean: mean deviation from the median [%]

## Table S.25. Clinical biochemistry: males -recovery-

Asterisks denote significance levels: n.s.: not significant (p > 0,05); *: 0,05 ≥ p > 0,01; **: 0,01 ≥ p > 0,001; ***: p ≤ 0,001. (Student ́s t-test two-tailed, unpaired); Median-Mean: mean deviation from the median [%]

## Table S.26. Hematology: females –recovery-

Asterisks denote significance levels: n.s.: not significant (p > 0,05); *: 0,05 ≥ p > 0,01; **: 0,01 ≥ p > 0,001; ***: p ≤ 0,001. (Student ́s t-test two-tailed, unpaired); Median-Mean: mean deviation from the median [%]

## Table S.27. Clinical biochemistry: females -recovery-

Asterisks denote significance levels: n.s.: not significant (p > 0,05); *: 0,05 ≥ p > 0,01; **: 0,01 ≥ p > 0,001; ***: p ≤ 0,001. (Student ́s t-test two-tailed, unpaired); Median-Mean: mean deviation from the median [%]

## Table S.28. Organ weight: males -main- (part 1)

Asterisks denote significance levels: n.s.: not significant (p > 0,05); *: 0,05 ≥ p > 0,01; **: 0,01 ≥ p > 0,001; ***: p ≤ 0,001 (Student ́s t-test two-tailed, unpaired).

## Table S.29. Organ weight: males -main- (part 2)

Asterisks denote significance levels: n.s.: not significant (p > 0,05); *: 0,05 ≥ p > 0,01; **: 0,01 ≥ p > 0,001; ***: p ≤ 0,001 (Student ́s t-test two-tailed, unpaired).

## Table S.30. Inductive statistical analysis: absolute and relative organ weights: males -main-

GraphPad Prism software was used for statistical analysis. Asterisks denote significance levels: n.s.: not significant (p > 0,05); *: 0,05 ≥ p > 0,01; **: 0,01 ≥ p > 0,001; ***: p ≤ 0,001 (Dunnett ́s post hoc t-test after 1-way ANOVA).

## Table S.31. Organ weight: females -main- (part 1)

Asterisks denote significance levels: n.s.: not significant (p > 0,05); *: 0,05 ≥ p > 0,01; **: 0,01 ≥ p > 0,001; ***: p ≤ 0,001 (Student ́s t-test two-tailed, unpaired).

## Table S.32. Organ weight: females -main- (part 2)

Asterisks denote significance levels: n.s.: not significant (p > 0,05); *: 0,05 ≥ p > 0,01; **: 0,01 ≥ p > 0,001; ***: p ≤ 0,001 (Student ́s t-test two-tailed, unpaired).

## Table S.33. Inductive statistical analysis: absolute and relative organ weights females -main-

GraphPad Prism software was used for statistical analysis. Asterisks denote significance levels: n.s.: not significant (p > 0,05); *: 0,05 ≥ p > 0,01; **: 0,01 ≥ p > 0,001; ***: p ≤ 0,001 (Dunnett ́s post hoc t-test after 1-way ANOVA).

## Table S.34. Inductive statistical analysis: absolute and relative organ weights females -main-

Heteroscedastic data (pos. Bartlett‘s test for equal variances) were analyzed using Kruskall-Wallis Test and Dunn‘s Multiple Comparison Test

## Table S.35. Organ weight: males -recovery-

Asterisks denote significance levels: n.s.: not significant (p > 0,05); *: 0,05 ≥ p > 0,01; **: 0,01 ≥ p > 0,001; ***: p ≤ 0,001 (Student ́s t-test two-tailed, unpaired).

## Table S.36. Organ weight: females -recovery-

Asterisks denote significance levels: n.s.: not significant (p > 0,05); *: 0,05 ≥ p > 0,01; **: 0,01 ≥ p > 0,001; ***: p ≤ 0,001 (Student ́s t-test two-tailed, unpaired).

## Fig. S.1. Decision tree for statistical analysis (schematic)

(*choice of ANOVA and Post-Hoc Test depending on the number of variables in the data set)
